# FUSION-Guided Hypothesis Development Leads to the Identification of *N*^6^,*N*^6^-Dimethyladenosine, a Marine-Derived AKT Pathway Inhibitor

**DOI:** 10.3390/md15030075

**Published:** 2017-03-15

**Authors:** Rachel M. Vaden, Nathaniel W. Oswald, Malia B. Potts, John B. MacMillan, Michael A. White

**Affiliations:** 1 Department of Cell Biology, University of Texas Southwestern Medical Center, Dallas, TX 75235, USA; Rachel.Vaden2@UTSouthwestern.edu (R.M.V.); Malia.Potts@StJude.org (M.B.P.); 2 Department of Biochemistry, University of Texas Southwestern Medical Center, Dallas, TX 75235, USA; Nathaniel.Oswald@UTSouthwestern.edu; 3 Cell & Molecular Biology Department, St. Jude Children’s Research Hospital, Memphis, TN 38105, USA

**Keywords:** dimethyladenosine, AKT, functional signature ontology

## Abstract

Chemicals found in nature have evolved over geological time scales to productively interact with biological molecules, and thus represent an effective resource for pharmaceutical development. Marine-derived bacteria are rich sources of chemically diverse, bioactive secondary metabolites, but harnessing this diversity for biomedical benefit is limited by challenges associated with natural product purification and determination of biochemical mechanism. Using Functional Signature Ontology (FUSION), we report the parallel isolation and characterization of a marine-derived natural product, *N*^6^,*N*^6^-dimethyladenosine, that robustly inhibits AKT signaling in a variety of non-small cell lung cancer cell lines. Upon validation of the elucidated structure by comparison with a commercially available sample, experiments were initiated to understand the small molecule’s breadth of effect in a biological setting. One such experiment, a reverse phase protein array (RPPA) analysis of >50 kinases, indicated a specific cellular response to treatment. In all, leveraging the FUSION platform allowed for the rapid generation and validation of a biological mechanism of action hypothesis for an unknown natural product and permitted accelerated purification of the bioactive component from a chemically complex fraction.

## 1. Introduction

**Figure 1:**
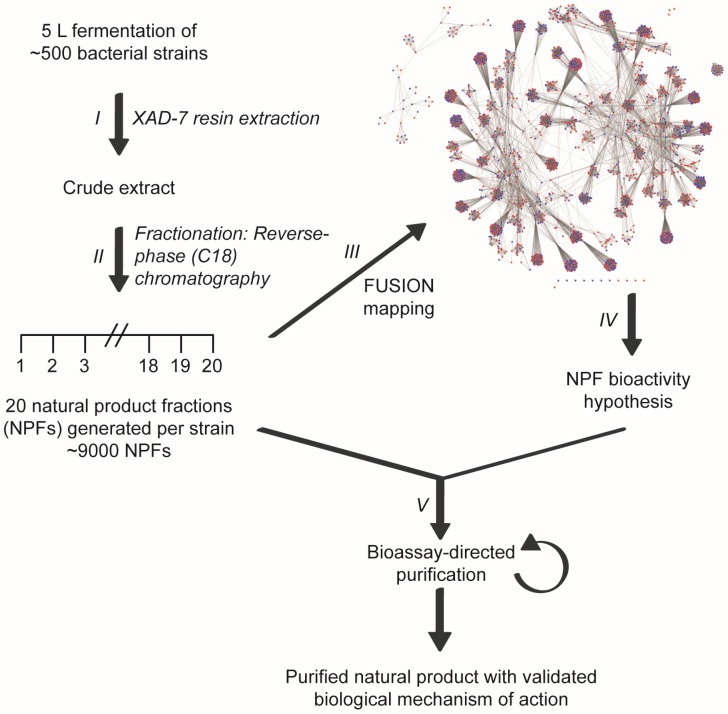
Linking small molecule-induced phenotypes with mechanisms of action using the Functional Signature Ontology (FUSION) platform. Crude extracts are generated from the fermentation broths of bacterial cultures (**I**) and subsequently fractionated using reverse-phase chromatography (**II**). Individual fractions are then assayed in a 6-gene reporter assay and the resulting expression signatures are mapped with those of other natural product fractions and genetic perturbagens (e.g., siRNAs) (**III**). This FUSION map allows for mechanism of action hypotheses to be generated for each natural product fraction based on its relationship with the gene signatures of known perturbagens (IV). Natural product fractions can then be tested in hypothesis-relevant bioassays to guide the fractionation process to a single, bioactive natural product (**V**).

The chemical diversity contained within microbial secondary metabolite pools can be employed to define new target space within biological processes that provoke human disease states. Historically, the challenge associated with this therapeutic development approach has been effectively connecting results obtained from biological, small molecule-induced phenotypic screens with biochemical mechanisms of action. Moreover, while a large number of new natural products with biological activity are reported yearly, a very small number are further evaluated from a mechanistic perspective. The chemically complex mixtures of natural products produced by microbes have also imposed constraints on the rate at which biologically active scaffolds can be identified [1]. To address these challenges and to help improve the pace of bioactive natural product discovery, we developed a high-throughput discovery platform that can link small molecule-induced phenotypes to molecular mechanisms of action [2]. This functional signature-based ontology (FUSION) platform connects biological perturbagens of known or inferred mechanisms of action (siRNAs, miRNAs, small molecule inhibitors) to those of unknown mechanisms of action (natural products, novel small molecules, siRNAs with gene targets of unknown function). The platform has been applied to a microbial natural products fraction library consisting ~9000 natural product fractions generated from the fermentation products of more than 500 marine-derived bacteria (Figure 1). For each cellular perturbagen, quantitative expression changes of a focused panel of endogenous reporter genes (*ACSL5*, *BNIP3L*, *ALDOC*, *LOXL2*, *BNIP3*, and *NDRG1*) are measured following exposure and these cellular responses are clustered and mapped to the cellular responses of the known perturbagens. Concordant responses allow for ‘guilt-by-association’ inferences to be made and mechanism of action hypotheses to be generated at scale. Herein, we report the identification and characterization of a marine-derived natural product whose biological mechanism of action includes the rapid attenuation of AKT signaling. Employing a FUSION-guided approach to hypothesis development significantly expedited the chemical isolation and purification process and facilitated the selection of an initial natural product fraction that was matched to our phenotype of interest.

## 2. Results

**Figure 2:**
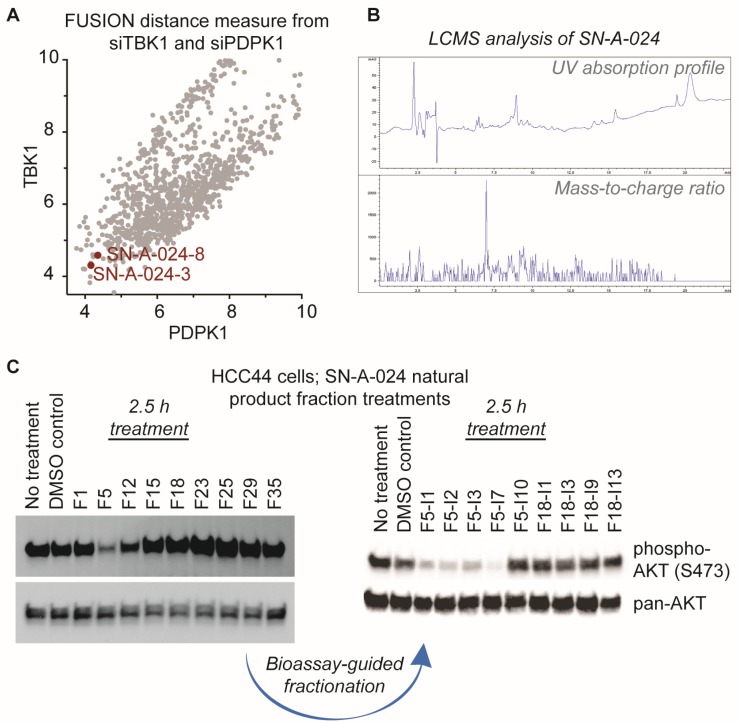
Identification of natural product fractions perturbing AKT signaling. (**A**) Evaluation of the FUSION map for natural product fractions clustering with AKT regulatory proteins, TBK1 and PDPK1 (TANK-binding kinase 1 and 3-phosphoinositide dependent protein kinase 1, respectively). Distance measure represents the Mahalanobis distance sum of both siTBK1 and siPDPK1; (**B**) Liquid chromatography-mass spectrometry (LCMS) analysis of the parent SN-A-024 natural product fraction; (**C**) Representative examples of the iterative, bioassay-guided fractionation process employed to identify the biologically active small molecule from the chemically complex SN-A-024 secondary metabolite pool. Each fraction was tested at a concentration of 100 micrograms per milliliter.

AKT signaling is central to a large number of functional cellular processes and is frequently dysregulated in diseases such as cancer [3,4,5,6,7,8]. With the overall goal of identifying natural products that target this signaling pathway, we evaluated the FUSION map for known perturbagens of AKT signaling; specifically, we assessed the intersection of the siRNA clades generated by siTBK1 and siPDPK1 (TANK-binding kinase 1 and 3-phosphoinositide dependent protein kinase 1, respectively) (Figure 2A). This approach was chosen because it has been established that TBK1 and PDPK1 (also known as PDK1) have direct regulatory roles in AKT activation [9,10,11]. We hypothesized that natural product fractions sharing similar, relatively small distance measures from both kinases within the FUSION map would perturb AKT signaling in a manner phenotypically similar to the known siRNAs. From the siTBK1-siPDK1 intersection, we identified two natural product fractions, SN-A-024-8 and SN-A-024-3, produced by the same strain of *Streptomyces coeruleoaurantiacus*. The strain (SN-A-024) was originally isolated from sediment samples obtained in West Plana Cay, Bahamas, and the fractions of secondary metabolites generated by the microbes were found to be chemically complex (Figure 2B). To test the FUSION-derived hypothesis that SN-A-024 produced small molecule modulators of AKT signaling, we cultured and re-fractionated the strain for biological testing. The resulting fractions were tested in HCC44 non-small cell lung cancer cells for their effect on AKT active site phosphorylation (serine 473) after 2.5 h of treatment. Of the nine fractions tested, one fraction (F5) was found to be unique in its ability to attenuate AKT phosphorylation at the S473 site (Figure 2C). Sub-fractionation of the F5 fraction by automated liquid chromatography (ISCO) followed by biological testing of the subsequent fractions further resolved a subset of chemical fractions with robust activity against AKT phosphorylation.

**Figure 3:**
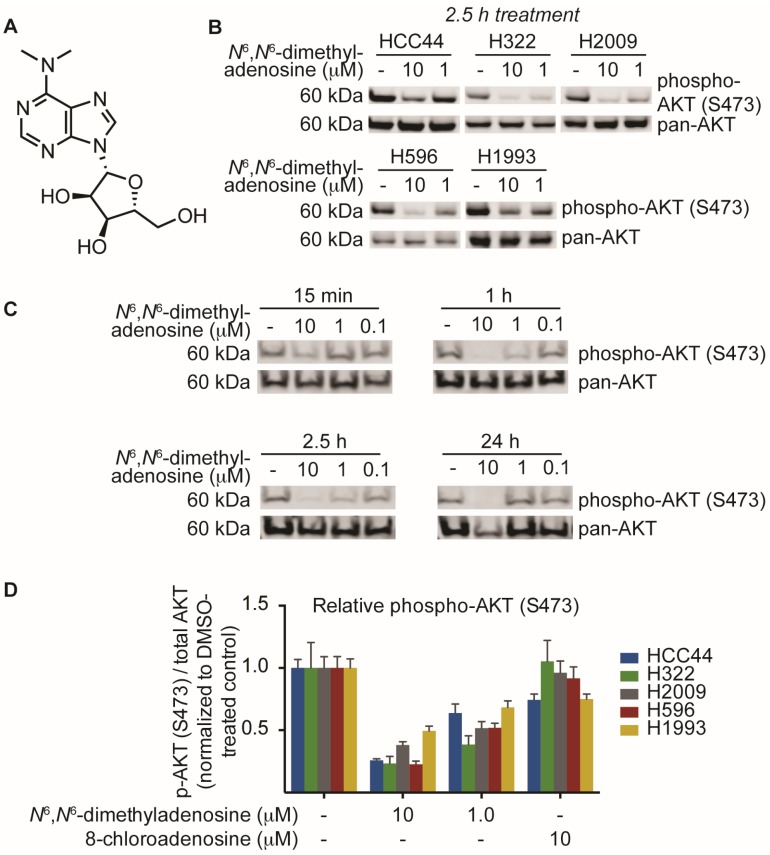
Identification and biological characterization of the active molecule identified from SN-A-024 using a FUSION-guided bioassay development strategy. (**A**) Structure of *N*^6^,*N*^6^-dimethyladenosine; (**B**) Evaluation of *N*^6^,*N*^6^-dimethyladenosine’s effect on the phosphorylation of AKT (S473) in five non-small cell lung cancer cell lines by immunoblot analysis; (**C**) Time course assessment of the effect of *N*^6^,*N*^6^-dimethyladenosine on AKT phosphorylation (S473) on HCC44 cells; (**D**) In-cell western analysis of AKT and AKT phosphorylation (S473). Cells were treated for one hour with the appropriate small molecule condition. The phospho-AKT (S473) data were first normalized to pan-AKT, then all data were normalized to their respective DMSO-treated controls to assess the overall change in phospho-AKT.

This process of biological phenotype-guided fractionation was iterated until a fraction enriched in a single molecule was isolated. High-resolution mass spectrometry (ESI-MS) analysis of the natural product afforded a mass/charge ratio consistent with the empirical formula C_12_H_17_N_5_O_4_ (HRMS *m/z* [M + H] 296.1358). Final structural elucidation was accomplished by coupling the high-resolution ESI-MS (HRMS) data with NMR analyses of the compound to reveal *N*^6^,*N*^6^-dimethyladenosine as the major metabolite (Figure 3A). After elucidation of the structure, we sought to validate our biological findings using a commercially available source of *N*^6^,*N*^6^-dimethyladenosine. Upon testing the small molecule against five non-small cell lung cancer cell lines, we found that commercial *N*^6^,*N*^6^-dimethyladenosine recapitulated SN-A-024’s effect on AKT signaling, attenuating S473 phosphorylation in all cell types tested at both 10 and 1 micromolar concentrations (Figure 3B). A time course assessment of *N*^6^,*N*^6^-dimethyladenosine’s effect on phospho-AKT in HCC44 cells established that the small molecule rapidly blunted AKT signaling, with only 15 min of treatment reducing levels by approximately half (Figure 3C).

Modified nucleoside analogs are commonly found in tRNA and rRNA in a variety of organisms and contribute to the structural diversity of RNA [12,13,14,15]. The nucleoside analog *N*^6^,*N*^6^-dimethyladenosine has been well-studied in the context of rRNA; the 3’-end of many species’ rRNA sequences contains two adjacent, dimethylated adenosine residues that participate in rRNA stability [16,17,18]. The protein complex responsible for the methylation of adenosine to form the *N*^6^,*N*^6^-dimethyl variant has not been fully characterized. The METTL3-METTL14 methyltransferase complex has been identified in mammalian cells as a biosynthetic mechanism by which the monomethylated variant *N*^6^-methyladenosine can be generated; interestingly though, the enzymes responsible for monomethylation do not appear to facilitate dimethylation events at the *N*^6^ position, suggesting functional distinctions in the methyltransferase components [19,20].

Several nucleoside analogs have been employed for therapeutic intervention in the clinic; vidarabine, a synthetic ribosyl-modified adenosine analog based upon isolates from a marine organism, has been used extensively as an antiviral therapeutic while 8-chloroadenosine and 8-aminoadenosine have been used as anti-cancer agents [21,22,23,24]. Despite the structural similarity between 8-chloroadenosine and the natural product isolate *N*^6^,*N*^6^-dimethyladenosine, 8-chloroadenosine has been reported to *increase* AKT S473 phosphorylation in the context of renal cell carcinoma [25,26]. We examined the possibility that the paradoxical differences observed between the two molecules may indicate lineage-specific sensitivity. Testing equimolar amounts of the two nucleoside analogs, we measured AKT phosphorylation by in-cell western in five lung cancer cell lines. The results indicated a clear separation in the activities of *N*^6^,*N*^6^-dimethyladenosine and 8-chloroadenosine: *N*^6^,*N*^6^-dimethyladenosine strongly inhibited AKT phosphorylation while 8-chloroadenosine elicited only a moderate effect in all cell lines tested (Figure 3D). These data suggest that the two nucleoside analogs differ in their mechanisms of action to some extent and more broadly, the results may indicate that specificity of nucleoside analog-protein binding events is higher than was initially considered.

**Figure 4:**
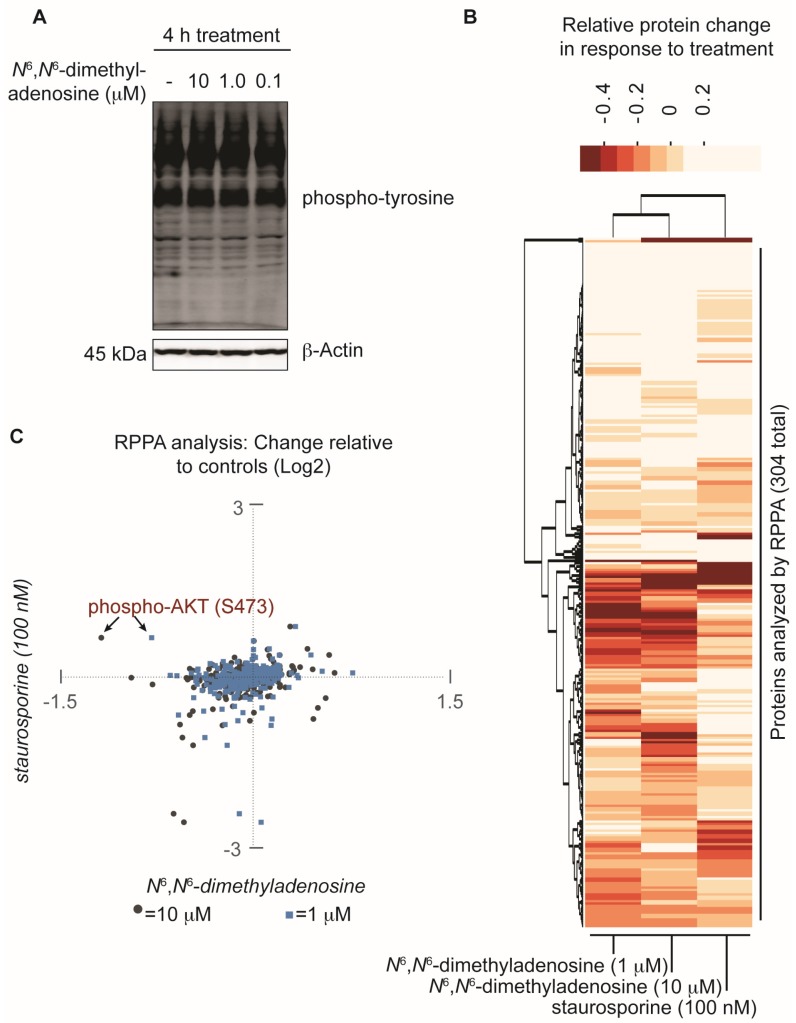
Assessment of the natural product *N*^6^,*N*^6^-dimethyladenosine’s effect on global protein perturbation. (**A**) Analysis of global phospho-tyrosine following a four hour treatment with *N*^6^,*N*^6^-dimethyladenosine in HCC44 cells; (**B**) Reverse phase protein array (RPPA) analysis of HCC44 cells treated for 2.5 h with either a vehicle control (DMSO), 10 micromolar *N*^6^,*N*^6^-dimethyladenosine, 1 micromolar *N*^6^,*N*^6^-dimethyladenosine, or 100 nanomolar staurosporine. The experiment was performed in duplicate and the replicates averaged. The Log2-transformed results were normalized to the DMSO vehicle control sample then analyzed with a 2-way unsupervised hierarchical cluster. The *y*-axis represents each antibody tested in the RPPA panel and the *x*-axis represents the experimental conditions examined; (**C**) Scatter plot comparison of RPPA values: staurosporine versus 10 micromolar *N*^6^,*N*^6^-dimethyladenosine (gray circles) and staurosporine versus 1 micromolar *N*^6^,*N*^6^-dimethyladenosine (blue squares). The RPPA data are Log2-transformed, replicate averaged, and control normalized.

Implementing a FUSION-directed strategy for the selection of a complex natural product fraction allowed for the rapid identification of SN-A-024’s biologically active metabolite from a complex mixture of chemicals. With our initial FUSION hypothesis confirmed and a purified molecule in hand, we next aimed to assess whether additional signaling pathways were impacted by *N*^6^,*N*^6^-dimethyladenosine. To address this, the nucleoside analog was first tested at three different concentrations against HCC44 cells and an immunoblot assay was used to measure global tyrosine phosphorylation. After four hours of treatment, the three *N*^6^,*N*^6^-dimethyladenosine-treated samples showed no change in total tyrosine phosphorylation compared to the control-treated sample, suggesting that the natural product’s effect on AKT resulted from specific pathway perturbations instead of global tyrosine kinome deregulation (Figure 4A). To further survey the effect of *N*^6^,*N*^6^-dimethyladenosine on other cellular signaling pathways, a reverse phase protein array (RPPA) experiment was designed and conducted using HCC44 cells (Supporting Information). Staurosporine, a broad-acting ATP-competitive kinase inhibitor, was included in the experiment to benchmark non-specific kinase inhibition effects in the natural product-treated samples. Notably, among the 304 antibodies tested in the RPPA panel, the largest *N*^6^,*N*^6^-dimethyladenosine-dependent change detected compared to control samples was AKT-S473 phosphorylation. Importantly, AKT-S473 phosphorylation was not inhibited by staurosporine. Globally, the RPPA experiment produced expected, dose-sensitive responses between the 10 micromolar and 1 micromolar *N*^6^,*N*^6^-dimethyladenosine-treated samples following normalization to the controls, while staurosporine displayed a distinct response (Figure 4B). Closer comparison of the staurosporine- versus *N*^6^,*N*^6^-dimethyladenosine-treated samples provided insight into the selective biological space targeted by each molecule (Figure 4C). Collectively, the RPPA results speak to the specificity of the marine-derived natural product and delineate its mechanism of action from those of promiscuous kinase effectors.

## 3. Discussion

The work presented herein describes the isolation and characterization of the marine-derived natural product *N*^6^,*N*^6^-dimethyladenosine, which was identified via an iterative fractionation process guided by the assessment of AKT phosphorylation at the S473 site. Nucleoside modifications can be found in all three domains of life and *N*^6^,*N*^6^-dimethyladenosine has specifically been studied in the context of rRNA function. Despite the ubiquitous presence of nucleoside modifications in single and multicellular organisms, we have only an inchoate understanding of their function at the present. The isolation and bioassay development process that led to the identification of *N*^6^,*N*^6^-dimethyladenosine was significantly expedited by first employing a FUSION-directed approach to generate a mechanism of action hypothesis. We initiated our study with the goal of identifying novel small molecules that could perturb the AKT signaling axis. Considering the dysregulated state of AKT signaling in many diseases, small molecule modulators targeting different pathway components are invaluable for expanding our basic understanding of disease mechanisms. Consistent with our initial hypothesis, we found that *N*^6^,*N*^6^-dimethyladenosine-treatment rapidly decreases the activity of AKT in a variety of non-small cell lung cancer cell lines. An RPPA panel consisting of 304 antibodies validated this finding and reinforced the biological basis for the connection made between SN-A-024 and siTBK1/siPDK1 in the FUSION map. The global perspective afforded by the RPPA experiment additionally suggested that the natural product’s mechanism of action exhibits some degree of specificity, despite its ostensibly ubiquitous scaffold. In all, this report details the successful implementation of the FUSION strategy to pair natural products with their biological modes of action. We report the effect of the natural product *N*^6^,*N*^6^-dimethyladenosine on AKT signaling and highlight the potential for this molecule as a tool for exploring new targetable biology surrounding AKT regulation. Given current efforts of the biological community to deploy natural product libraries in large-scale, cell-based phenotypic screens, we anticipate the FUSION platform will also have general utility for stratification of hits from these screens into biological “complementation groups” which will, in turn, allow for accelerated annotation of the biological modes-of-action of molecules with bioactivities of interest.

## 4. Materials and Methods

### 4.1. Collection and Phylogenetic Analysis of Strain SNA-024

The marine-derived bacterium strain SNA-024 was isolated from a sediment sample collected from West Plana Cay, Bahamas. Bacterial spores were collected via a stepwise centrifugation as follows: 2 grams of sediment was dried over 24 h in an incubator at 35 °C and the resulting sediment was added to 10 mL sH_2_O containing 0.05% Tween 20. After vigorous vortex for 10 min, the sediment was centrifuged at 18,000 rpm for 25 min (4 °C) and the resulting spore pellet was collected. The resuspended spore pellet (4 mL sH_2_O) was plated on an acidified Gause media, giving rise to individual colonies of SNA-024 after 2 weeks. Analysis of the 16S rRNA sequence of SNA-024 revealed 98% identity to *Streptomyces coeruleoaurantiacus*.

### 4.2. Cultivation and Extraction

Bacterium SNA-024 was cultured in 15 × 2.8 L Fernbach flasks each containing 1 L of seawater-based medium (10 grams starch, 4 grams yeast extract, 2 grams peptone, 1 gram CaCO_3_, 40 milligrams Fe_2_(SO_4_)_3_·4H_2_O, 100 milligrams KBr) and shaken at 200 rpm at 27 °C. After seven days of cultivation, sterilized XAD-7-HP resin (20 g/L) was added to absorb the organic products, and the culture and resin were shaken at 200 rpm for 2 h. The resin was filtered through cheesecloth, washed with deionized water, and eluted with acetone. The acetone-soluble fraction was dried in vacuo to yield 11.4 grams of extract.

### 4.3. Purification of a Fraction Enriched in N^6^,N^6^-Dimethyladenosine

Crude extract (11.4 grams) was separated into nine fractions by reverse phase chromatography (C18) using a stepwise gradient, with MeOH/H_2_O (20%–100%). The biologically active fraction (F5, 91.4 milligrams) was further purified using an automated reversed phase chromatography (ISCO, RediSep Rf Gold 30 grams C18, 35 mL/min) using a gradient solvent system from 10% to 100% MeOH:H_2_O over 25 min, and 30 fractions (F5-I1–F5-I30) were collected. Fractions were combined based on LC-MS profile similarity to a total of five fractions (F5-I1, F5-I3, F5-I5, F5-I7, and F5-I9). Biologically active F5-I7 (5.3 milligrams) was further purified by reverse-phase HPLC (Phenomenex Luna, C18, 250 × 10.0 mm, 5 μm, 2.5 mL/min) using a gradient solvent system, acetonitrile/water with 0.1% formic acid (10%–100%) over 20 min with five fractions collected. Fraction F5-I7-1 (~0.5 milligrams, *t*_R_ = 7.0 min) was biologically active and used for structural characterization (HRMS and NMR), and biological assays. As described below, the inseparable mixture contained in fraction F5-I7-1 was analyzed by HRMS and NMR to determine the major component.

### 4.4. NMR Characterization

High-resolution ESI-MS (HRMS) analysis of the enriched fraction F5-I7-1 gave *m/z* 296.1352 [M + H]⁺ for the major metabolite consistent with a molecular formula of C_12_H_17_N_5_O_4_ (calculated for C_12_H_18_N_5_O_4_, 296.1358). ^1^H-NMR data indicated that the major metabolite was a nucleoside in nature with two aromatic signals and signals diagnostic of a glucoside. Analysis of the molecular formula and the diagnostic NMR signals suggested a structure consistent with *N*^6^,*N*^6^-dimethyladenosine. Due to fraction F5-I7-1 material constraints, commercially available *N*^6^,*N*^6^-dimethyladenosine (ChemBridge Corporation, San Diego, CA, USA) was used to directly compare the ^1^H NMR. ^1^H-NMR comparison of commercial *N*^6^,*N*^6^-dimethyladenosine with the enriched fraction F5-I7-1 indicated that the major metabolite was *N*^6^,*N*^6^-dimethyladenosine (Supplementary Table S1, Supplementary Figure S1).

### 4.5. Reagents and Antibodies

HCC44, H1993, H322, H1993, and H596 cells were cultured in Dulbecco’s Modified Eagle Medium (DMEM) supplemented with 5% fetal bovine serum and penicillin/streptomycin. Cells were maintained in a humidified incubator at 37 °C in 5% CO_2_. 8-chloroadenosine was obtained from Santa Cruz Biotechnology. For immunoblot analyses, the following antibodies were used: pan-AKT (Cell Signaling Technologies #4691; 1:1000 dilution), phospho-AKT (S473) (Cell Signaling Technologies #4060; 1:2000 dilution), β-Actin (Cell Signaling Technologies #3700; 1:1000 dilution), and phospho-Tyrosine (Cell Signaling Technologies #9411; 1:2000 dilution). All antibody dilutions were made in Odyssey Blocking Buffer (Li-Cor Biosciences, Lincoln, NE, USA). Western membrane blocking was also accomplished with Odyssey Blocking Buffer. Immunoblots imaged with the Li-Cor Odyssey imaging system were incubated with the appropriate Li-Cor secondary antibodies at 1:10,000 before imaging. For experiments utilizing commercially sourced *N*^6^,*N*^6^-dimethyladenosine, the small molecule used was obtained from ChemBridge (San Diego, CA, USA).

### 4.6. In-Cell Western

For in-cell western experiments, cells were seeded in 96-well plates at 20,000 cells per well and treated with the appropriate drug 16 h after seeding. One hour after the start of treatment, the media was removed and cells were fixed with 4% paraformaldehyde (*w/v*) for 10 min at room temperature. Following the removal of the formaldehyde solution, permeabilization was accomplished by incubating the cells for 30 min with 0.1% Triton-X (*v/v*) in 1× PBS. The wells were blocked for one hour at room temperature with Odyssey Blocking Buffer, then incubated with the primary antibodies overnight at 4 °C. The antibodies used included pan-AKT (Cell Signaling Technologies #4691; 1:400 dilution) and phospho-AKT (S473) (Cell Signaling Technologies #4060; 1:200 dilution). Following the overnight incubation, wells were incubated with the appropriate Li-Cor secondary antibodies at 1:800 before imaging on the Li-Cor Odyssey imaging system.

### 4.7. Reverse Phase Protein Array

For reverse phase protein array (RPPA) experiments, HCC44 cells were seeded in duplicate in 6-well plates and treated with the appropriate compound for 2.5 h. Following the completion of treatment, cell lysates were collected and prepared per the MD Anderson RPPA Core Facility submission guidelines. Briefly, the media was removed from the treated cells, the wells were washed two times with ice cold 1× PBS, and ice cold lysis buffer was added to the plate (1% Triton X-100 (*v/v*), 50 mM 4-(2-hydroxyethyl)-1-piperazineethanesulfonic acid) (HEPES), pH 7.4, 150 mM NaCl, 1.5 mM MgCl_2_, 1 mM ethylene glycol-bis(β-aminoethyl ether)-*N*,*N*,*N*',*N*'-tetraacetic acid (EGTA), 100 mM NaF, 10 mM Na pyrophosphate, 1 mM Na_3_VO_4_, 10% glycerol (*v/v*), and freshly added protease and phosphatase inhibitors). The plate was incubated on ice for 20 min with the lysis buffer, shaking every 5 min. The lysates were transferred to a microcentrifuge tube and centrifuged for 10 min at 14,000 rpm at 4 °C. The supernatant was collected and protein concentration quantified. The lysates were combined with 4× sample buffer (40% Glycerol, 8% Sodium dodecyl sulfate (SDS), 0.25 M Tris-HCl, pH 6.8) and frozen at −80 °C. The RPPA experiment and subsequent data analysis was performed by MD Anderson RPPA Core Facility. Log2 transformed values were used for analysis and plotting; 2-way hierarchical cluster analysis was performed with R, version 3.2.3 using the ‘stats’ package and heatmap function.

## Supplementary Materials

The following are available online at www.mdpi.com/1660-3397/15/3/75/s1: Supplemental Information–RPPA data.xlsx and Supplemental Information–Methods.pdf.
