# Supplementary material for: FUSION-Guided Hypothesis Development Leads to the Identification of N6,N6-Dimethyladenosine, a Marine-Derived AKT Pathway Inhibitor
Source: Mar Drugs. 2017 Mar 15;15(3):75. doi: 10.3390/md15030075 (PMC5367032; doi:10.3390/md15030075)
Supplement: Supplementary file 1 [file marinedrugs-15-00075-s001.pdf]

Supplemental Information File

General NMR, LC-MS and HPLC procedures

^{1}H and 2D experiments were recorded at a 600MHz using DMSO-d_6 in a Varian spectrometer and chemical shifts were recorded based on the corresponding solvent signal (δ_H 2.50 ppm for DMSO-d_6).

High resolution ESI-TOF (HRMS) mass spectra were provided by the Scripps Research Institute, La Jolla, CA. Low-resolution LC/ESI-MS data were measured using an Agilent 1200 series LC/MS system -with a reverse-phase C18 column (Phenomenex Luna, 150mm X 4.6 mm, 5 μm) at a flow rate of 0.7 mL/min. Semi-preparative HPLC was performed on an Agilent 1200 series, using a C18 column (Phenomenex Luna, 250 mm X 10.0 mm, 5μm).

Collection and phylogenetic analysis of strain SNA-024

The marine-derived bacterium strain SNA-024 was isolated from a sediment sample collected from West Plana Cay, Bahamas. Bacterial spores were collected via a stepwise centrifugation as follows: 2 g of sediment was dried over 24 hr in an incubator at 35˚ and the resulting sediment added to 10 mL sH_2O containing 0.05% Tween 20. After vigorous vortex for 10 min, the sediment was centrifuged at 18000 rpm for 25 min (4˚ C) and the resulting spore pellet collected. The resuspended spore pellet (4 mL sH_2O) was plated on an acidified Gause media, giving rise to individual colonies of SNA-024 after 2 weeks. Analysis of the 16S rRNA sequence of SNA-024 revealed 98% identity to *Streptomyces coeruleoaurantiacus*.

Cultivation and Extraction

Bacterium SNA-024 was cultured in 15 X 2.8 L Fernbach flasks each containing 1 L of seawater-based medium (10 g starch, 4 g yeast extract, 2 g peptone, 1 g $CaCO_3$, 40 mg $Fe_2(SO_4)_3{\bullet}4H_2O$, 100 mg KBr) and shaken at 200 rpm at 27 °C. After seven days of cultivation, sterilized XAD-7-HP resin (20 g/L) was added to absorb the organic products, and the culture and resin were shaken at 200 rpm for 2 h. The resin was filtered through cheesecloth, washed with deionized water, and eluted with acetone. The acetone-soluble fraction was dried *in vacuo* to yield 11.4 g of extract.

Purification of *N*6,*N*6-dimethyladenosine

Crude extract (11.4g) was separated into 9 fractions by reverse phase chromatography (C18) using a stepwise gradient, with MeOH /H_2O (20%-100%). The biologically active fraction (**F5**, 91.4mg) was further purified using an automated reversed phase chromatography (ISCO, RediSep Rf Gold® 30 g C18, 35 ml/min) using a gradient solvent system from 10%-100% MeOH:H_2O over 25 min and 30 fractions (F5-I1 – F5-I30) were collected. Fractions were combined based on LC-MS profile similarity to a total of 5 fractions (F5-I1, F5-I3, F5-I5, F5-I7 and F5-I9). Biologically active F5-I7 (5.3mg) was further purified by reverse-phase HPLC (Phenomenex Luna, C18, 250 X 10.0 mm, 5 μm, 2.5 mL/min) using a gradient solvent system, acetonitrile/water with 0.1% formic acid (10%-100%) over 20 minutes with five fractions collected. Fraction F5-I7-1 (~0.5 mg, t_R = 7.0 min) was biologically active and used for structural characterization (HRMS and NMR), and biological assays.

NMR characterization

High-resolution ESI-MS (HRMS) analysis of the enriched fraction **F5-I7-1** gave *m/z* 296.1352 $[M + H]^+$ for the major metabolite consistent with a molecular formula of $C_{12}H_{17}N_5O_4$ (calcd for $C_{12}H_{18}N_5O_4$, 296.1358). ^1H NMR data indicated that the major metabolite was a nucleoside in nature with two aromatic signals and signals diagnostic of a glucoside. Analysis of the molecular formula and the diagnostic NMR signals suggested a structure consistent with N^6,N^6-dimethyladenosine. Due to material constraints of fraction **F5-I7-1** and the inability to get a pure sample, we ordered commercial N^6,N^6-dimethyladenosine (ChemBridge™, ChemBridge Corporation, San Diego, CA, USA) to directly compare the ^1H NMR.

^1H NMR comparison of commercial N^6,N^6-dimethyladenosine with the enriched fraction **F5-I7-1** indicated that the major metabolite is N^6,N^6-dimethyladenosine (Supplemental Table 1). Comparison of the aromatic H-2 at δ_H at 8.21(s), and H-8 at δ_H at 8.37(s), the anomeric H-1′ at δ_H at 5.90 (d, *J* = 6.0Hz), and remaining ribulose signals H-2′ at δ_H 4.56 (ddd, *J* = 5.6, 6.0, 6.2), H-3′ at δ_H 4.14 (ddd, *J* = 4.8, 5.0, 6.2), H-4′ at δ_H 3.96 (ddd, *J* = 3.6, 3.7, 4.0), H-5′ at δ_H 3.67(m) and δ_H 3.55(m) with exchangeable protons 2′-OH at δ_H 5.2, 3′-OH at δ_H 5.3 and 4′-OH at δ_H 5.5 unequivocally establish the major metabolite in enriched fraction **F5-I7-1** as N^6,N^6-dimethyladenosine (Supplemental Table 1, Supplemental Figure 1-3). Analysis of the remaining ^1H signals (δ_H 8.15 to δ_H 8. 40 ppm and δ_H 3.50 to 6.10 ppm) in **F5-I7-1** revealed that the other metabolites are related adenosine analogs, potentially a diastereomers of N^6,N^6-dimethyladenosine.

Supplemental Table 1. 1D ^1H NMR data for **F5-I7-1**, N^6,N^6-dimethyladenosine and adenosine.

No.	δ_H, mult. (*J* in Hz) **F5-I7-1**	δ_H, mult. (*J* in Hz) N^6,N^6-dimethyladenosine	δ_H, mult. (*J* in Hz) adenosine
1	-	-	-
2	8.21 *s**	8.21 *s*	8.13 *s*
3	-	-	-
4	-	-	-
5	-	-	-
6	-	-	-
7	-	-	-
8	8.37 *s**	8.37 *s*	8.34 *s*
9	-	-	-
1'	5.90 *d (6.0)**	5.90 *d (6.0)*	5.87 *d (6.2)*
2'	4.56 *ddd (5.0)**	4.56 *ddd (5.6, 6.0, 6.2)*	4.61 *ddd (5.1, 6.2, 6.3)*
3'	4.14 *ddd (3.5)**	4.14 *ddd (4.8, 5.0, 6.2)*	4.14 *ddd (3.0, 4.6, 5.1)*
4'	3.96 *ddd (3.6, 3.7, 3.8)**	3.96 *ddd (3.6, 3.7, 4.0)*	3.96 *ddd (3.0, 3.6, 3.7)*
5'	3.67 *m**	3.67 *ddd (4.0, 5.0, 12.1)*	3.67 *ddd (3.6, 4.4, 12.1)*
	3.55 *m**	3.55 *ddd (3.7, 7.0, 12.1)*	3.55 *ddd (3.7, 7.2, 12.1)*
2'OH	*5.5 m**	5.46 *d (6.3)*	5.43 *d (6.3)*
3'OH	*5.2 m**	5.19 *d (4.7)*	5.17 *d (4.6)*
5'OH	*5.3 m**	5.38 *dd (4.5, 7.1)*	5.41 *dd (4.4, 7.2)*
*N*6-Me	*3**	*3.33 s*	-

* overlap with other metabolites

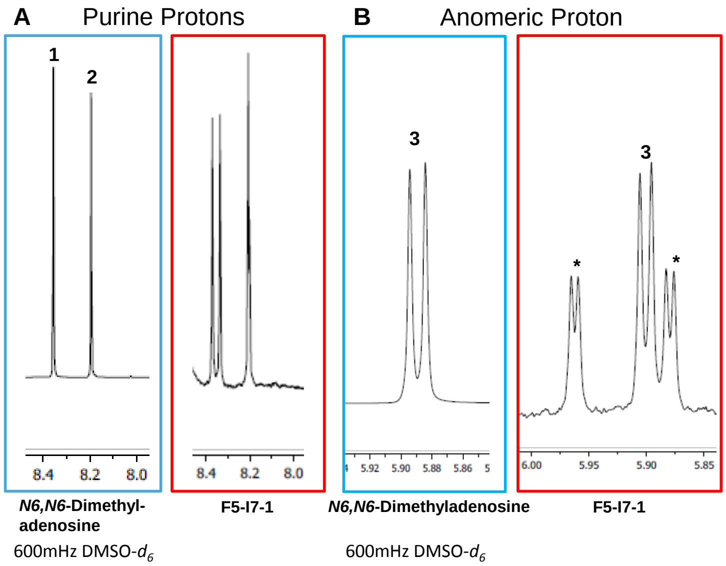

Supplemental Figure 1. 1D ^{1}H NMR comparison of **F5-I7-1** and N^6,N^6-dimethyladenosine H2, H8 and H1' protons.

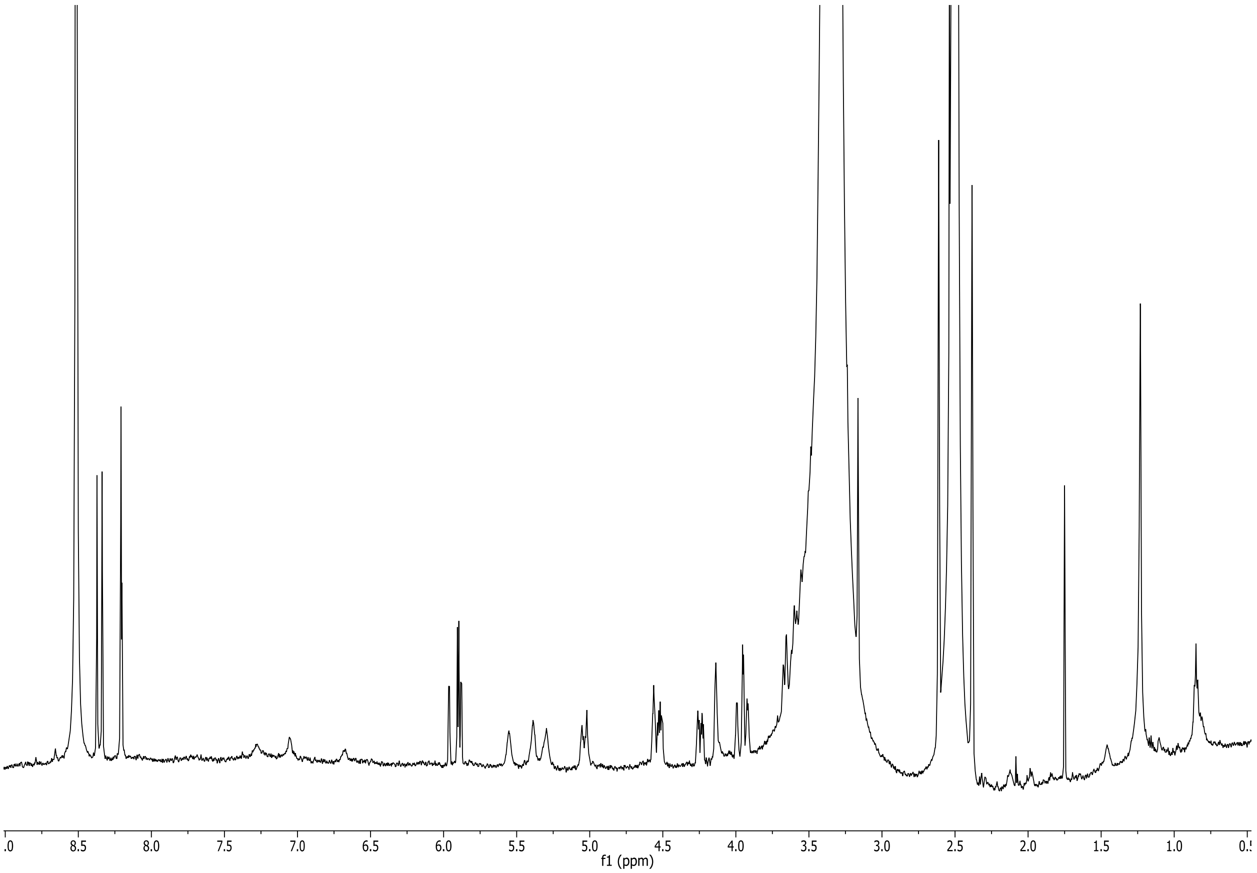

Supplemental Figure 2. 1D proton NMR of **F5-I7-1** using 600MHz in DMSO-d_6.

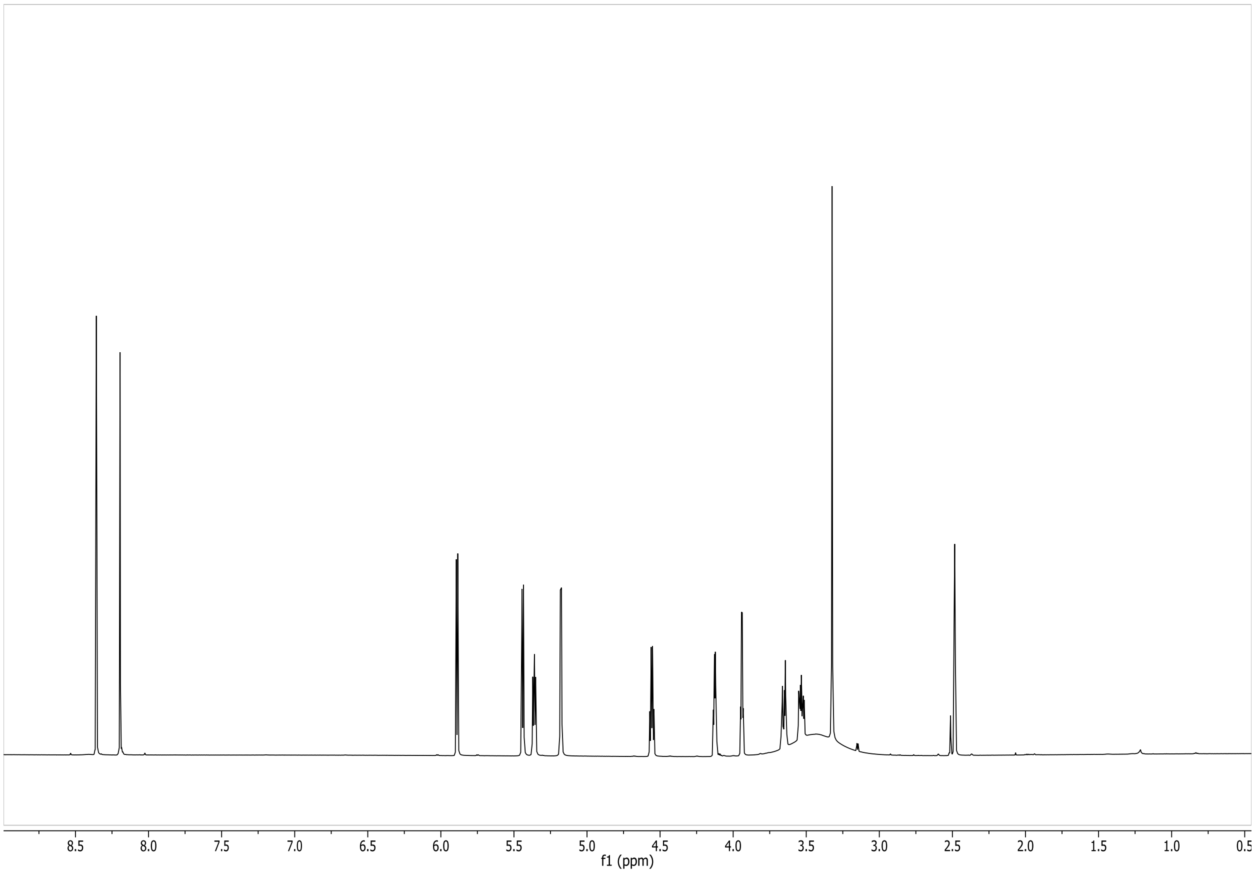

Supplemental Figure 3. 1D proton NMR of *N*^{6},*N*^{6}-dimethyladenosine at 600MHz in DMSO-*d*$_6$.

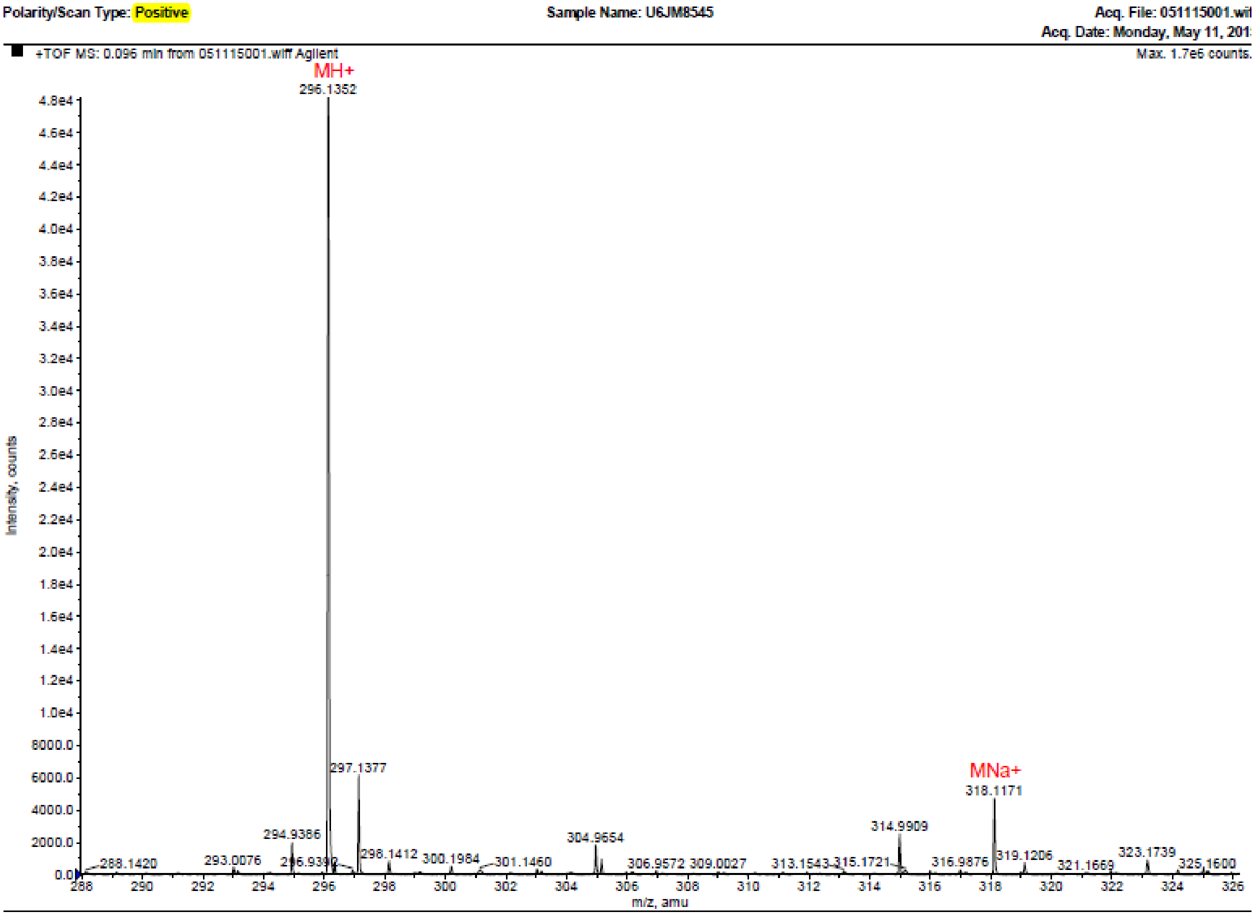

Supplemental figure 4. HRMS of **F5-I7-1** showing peak at m/z 296.1352 $[M + H]^{+}$.
